# Simultaneous detection of ethambutol and pyrazinamide with IL@CoFe_2_O_4_NPs@MWCNTs fabricated glassy carbon electrode

**DOI:** 10.1038/s41598-020-70263-z

**Published:** 2020-08-11

**Authors:** Rajasekhar Chokkareddy, Suvardhan Kanchi, Inamuddin

**Affiliations:** 1 Department of Chemistry, Durban University of Technology, Durban, 4000 South Africa; 2 Department of Chemistry, Sambhram Institute of Technology, M.S. Palya, Jalahalli East, Bengaluru, 560097 India; 3 Chemistry Department, Faculty of Science, King Abdulaziz University, Jeddah, 21589 Saudi Arabia; 4 Advanced Functional Materials Laboratory, Department of Applied Chemistry, Faculty of Engineering and Technology, Aligarh Muslim University, Aligarh, 202002 India

**Keywords:** Bioanalytical chemistry, Nanoparticles

## Abstract

For the first time, we report a novel electrochemical sensor for the simultaneous detection of ethambutol (ETB) and pyrazinamide (PZM) using 1-ethyl-3-methylimidazolium tetrafluoroborate ([Emim][BF_4_]) ionic liquid (IL) assimilated with multiwalled carbon nanotubes (MWCNTs) decorated cobalt ferrite nanoparticles (CoFe_2_O_4_NPs) on the surface of glassy carbon electrode (GCE). The surface morphological and electrochemical properties of the IL@CoFe_2_O_4_NPs@MWCNTs was characterized with X-ray diffraction (XRD), transmission electron microscope (TEM), thermogravimetric analysis (TGA), fourier transform infrared spectroscopy (FTIR) and cyclic voltammetry (CV), differential pulse voltammetry (DPV) respectively. Moreover, the obtained results of CV demonstrated that the 9-folds enhancement in the electrochemical signals was achieved with IL@CoFe_2_O_4_NPs@MWCNTs@GCE compared to that of a bare GCE. Additionally, the simultaneous electrochemical detection of ETB and PZM was successfully accomplished using IL@CoFe_2_O_4_NPs@MWCNTs over a wide-range of concentration with good limit of detection (3S/m) of 0.0201 and 0.010 μM respectively. The findings of this study identify IL@CoFe_2_O_4_NPs@MWCNTs@GCE has promising abilities of simultaneous detection of ETB and PZM in pharmaceutical formulations.

## Introduction

**Figure 1:**
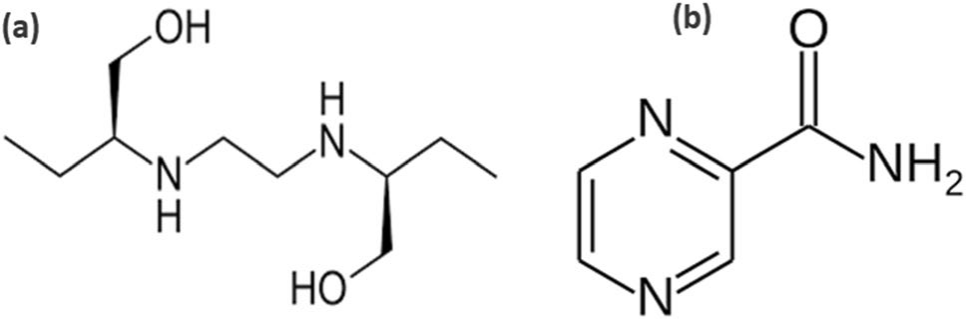
Chemical structures. (**a**) Ethambutol. (**b**) Pyrazinamide.

Tuberculosis (TB) is a disease caused by a germ called *Mycobacterium tuberculosis* that is spread from person to person through the air. TB usually affects the lungs, but it can also affect other parts of the body, such as the brain, the kidneys, or the spine^1,2^. Recent World Health Organization reports suggest that 9.6 million people were infected with 1.5 million deaths every year worldwide^2^. It is found that different tuberculosis strains have developed resistance to the drugs that use during the treatment process. In order to eradicate the growth of tuberculosis, ethambutol hydrochloride (ETB), pyrazinamide (PZM) drugs have been extensively used in combination with a fixed doses of isoniazid and rifampicin as shown in Fig. 1a,b. The use of ETB and PZM for specific period of time has shortened the treatment time, however few side effects including anorexia, nausea, liver injury, fever, vomiting, sideroblastic anemia and severe hepatic damages^3–5^ has been noted. Therefore, it is necessary to have a sensitive, selectivity and robust analytical method to monitor the concentration of ETB and PZM in biological fluids to avoid side effects in the TB patients.

The survey of literature reports suggests that several analytical techniques have been reported for the separation and detection of ETB and PZM, including gas chromatography^6^, high performance liquid chromatography^7^, capillary electrophoresis^8^, gas chromatography–mass spectrometry^9^, chemiluminescence^10^ and spectrophotometry^11^. Although these techniques have significant scientific merits such as good sensitivity and low detection limits, but they require long treatment procedures by the qualified operator with high cost implications. Alternatively, electrochemical procedures offers imperative advantages, such as simplicity with cost effectiveness, rapid response, selectivity and high sensitivity^12,13^ in the detection of analyte in various sample matrices. The fabrication of the GCE by nanoparticles is an active method of improving the catalytic action of the sensors, resulting in the amplification of the electrochemical signals. The MWCNTs is a unique smart nanomaterial adopted in the fabrication of electrode surfaces due to their high flexibility, high tensile strength, elasticity and high conductivity of the electrons^14,15^. Thus, the MWCNTs was used as a fundamental electrode coating material in the present study.

Recently, metallic and transition metal oxide nanoparticles especially spinel ferrite MFe_2_O_4_ has growing the interest of researchers in the development of ultrasensitive electrochemical biosensors with multifaceted applications ranging from environmental to point-of-care applications, whereas magnetic and electrical conducting nature of the MFe_2_O_4_ nanocomposite depends on the M^2+^ cation^16,17^. The cations such as Co, Mg, Cu, Ni, Fe, Zn and Mn were often used metals in the nanocomposites^18^. Generally, spinels ferrite substance CoFe_2_O_4_ has significant features including magnetic, electronic, energy storage and analytical biochemical applications^19^. Nowadays ionic liquids (ILs) based electrochemical techniques with ion-selective sensors has attracted scientists in the fabrication of biosensor devices due to its improved lifetime, stability, promotes rapid electron transfer and sensitivity. The good catalytic ability together with the facile experimental methodologies significantly promotes the application of ILs-CNs based microelectrodes in the electrochemical sensing of different analytes^20–22^.

In this study, a selective and sensitive method was established to develop a robust electrochemical sensing platform with IL@CoFe_2_O_4_NPs@MWCNTs@GCE. To the best of our knowledge, IL@CoFe_2_O_4_NPs@MWCNTs@GCE is the first report on its own for the simultaneous detection of ETB and PZM and successfully applied to the pharmaceutical formulations.

## Experimental

### Chemicals and reagents

MWCNTs (O.D.-L 6–9 nm to 5 μM), ethambutol (ETB), 1-ethyl-3-methylimidazolium tetrafluoroborate ([Emim][BF_4_]) ionic liquid (IL) and pyrazinamide (PZM) were purchased from Sigma Aldrich (Durban, RSA). Sulphuric acid (H_2_SO_4_), disodium hydrogen orthophosphate (Na_2_HPO_4_), ethanol (C_2_H_6_O), sodium hydroxide (NaOH), hydrochloric acid (HCl), sodium dihydrogen orthophosphate (NaH_2_PO_4_), nitric acid (HNO_3_), ferric chloride (FeCl_3_·6H_2_O), N, Nʹ- dimethyl formamide (DMF) and cobalt chloride (CoCl_2_·6H_2_O) were purchased from the Capital Lab Suppliers (Durban, RSA). All the chemicals were used as received without any further purification.

### Instrumentation

All electrochemical studies were performed with 797VA computrace system (Metrohm Herisau, Switzerland) with conventional three-electrode system including Ag/AgCl (3 M KCl), GCE and platinum wire as reference, working and auxiliary electrodes respectively. Fourier transformation infrared (FTIR) characterization was performed using Varian 800 FTIR scimitar series (by SMM instruments). TGA/DSC analysis was conducted with STAR^e^ system (model: 1 SF/1346) from METTLER TOLEDO. A CRISON digital micro pH meter with an accuracy of ± 0.1 was used for the pH adjustments. A TEM (model: JEM 2100 with a Lab 6 emitter) was used to evaluate the surface morphology of the nanocomposite. Furthermore, sonication was performed with a LABCON 5019 U model throughout the study.

### Synthesis of CoFe_2_O_4_ nanoparticles

CoFe_2_O_4_ nanoparticles (CoFe_2_O_4_NPs) were synthesized by co-precipitation method with slight modification^23^, 0.2 M (50 mL) of FeCl_3_ and a 0.4 M (50 mL) of CoCl_2_ were mixed in deionized distilled water. 4 M (50 mL) of NaOH was prepared and dropwise added to the salt solution. After adding of NaOH the pH of the solution was continuously observed. The solution mixture was continuously stirred using a magnetic stirrer until it reach pH 12. Additionally, a definite volume (2 mL) of oleic acid was added and subjected to heating for 60 min at 90 °C, resulting in a brown colour precipitate. The obtained precipitate was given several washings with deionized distilled water followed by the ethanol to eliminate free Na^+^, Cl^−^ as well as other surfactant and dried for 15 h at 90 °C. Finally, the obtained solid NPs material was then grinded into a fine powder for further use.

### Electrode modification

Before the fabrication process, the GCE was polished to a mirror like surface with 0.3 μm alumina slurry. Then after, the electrode was washed with deionized distilled water for five times. Thereafter, the GCE was sonicated with an aqueous solution of deionized distilled water and ethanol (1/1, v/v) for 15 min. The MWCNTs suspension was made by the previous described procedure with slight amendment^24^. Firstly, 10 mg of MWCNTs was dispersed in 20 mL of DMF and then the suspension was sonicated for about 1 h at 40 °C, resulting in the formation of a black precipitate which was used for the modification of GCE^2^. Secondly, 15 mg of MWCNTs and 15 mg of CoFe_2_O_4_NPs were dispersed into 30 mL of DMF solution and subjected to sonication for 5 h to form a thick black precipitate of CoFe_2_O_4_NPs decorated MWCNTs nanocomposite. Furthermore, GCE was fabricated by dropping 5 µL of CoFe_2_O_4_NPs@MWCNTs nanocomposite and dried at 60 °C for 5 min. Finally, CoFe_2_O_4_NPs@MWCNTs@GCE was dipped into IL solution for 90 min and kept undisturbed at 4 °C for about 10 min for complete immobilization process and then allowed to dry at ambient temperature for 5 min. The obtained electrode was designated as IL@CoFe_2_O_4_NPs@MWCNT@GCE.

### Real sample preparation

Five of each commercial brands were obtained from a local dispensary, exactly weighed, and then grounded to a fine residue with mortar and pestle. The obtained powder was dissolved in 10 mL deionized distilled water and sonicated for 20 min. The resulted mixture was filtered with a Whatman no: 1 paper and 10 mL of the successive solution was then transferred into a 15 mL standard flask and dilute with phosphate buffer solution.

## Results and discussion

### Characterization of IL@CoFe_2_O_4_NPs@MWCNT@GCE

**Figure 2:**
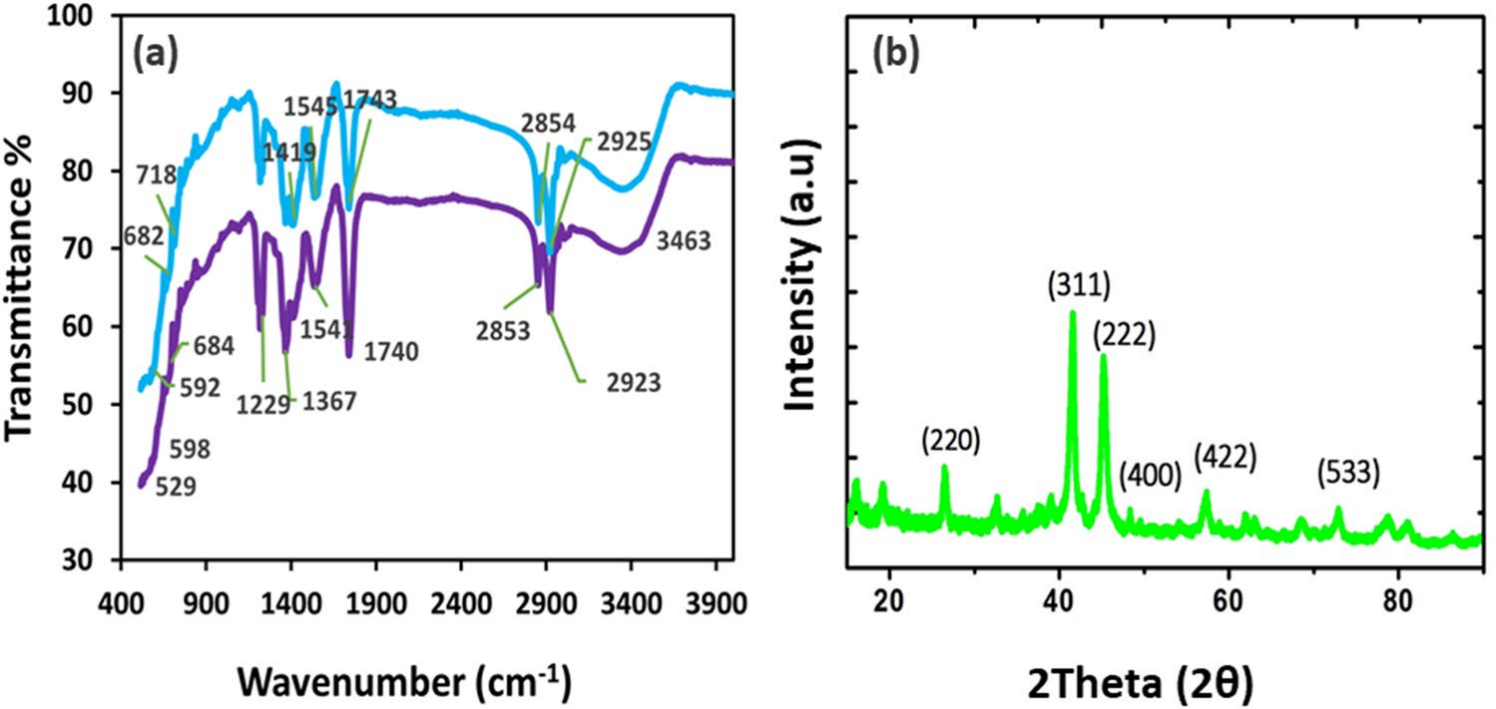
The characterization of CoFe_2_O_4_NPs with (**a**) FTIR and (**b**) XRD.

**Figure 3:**
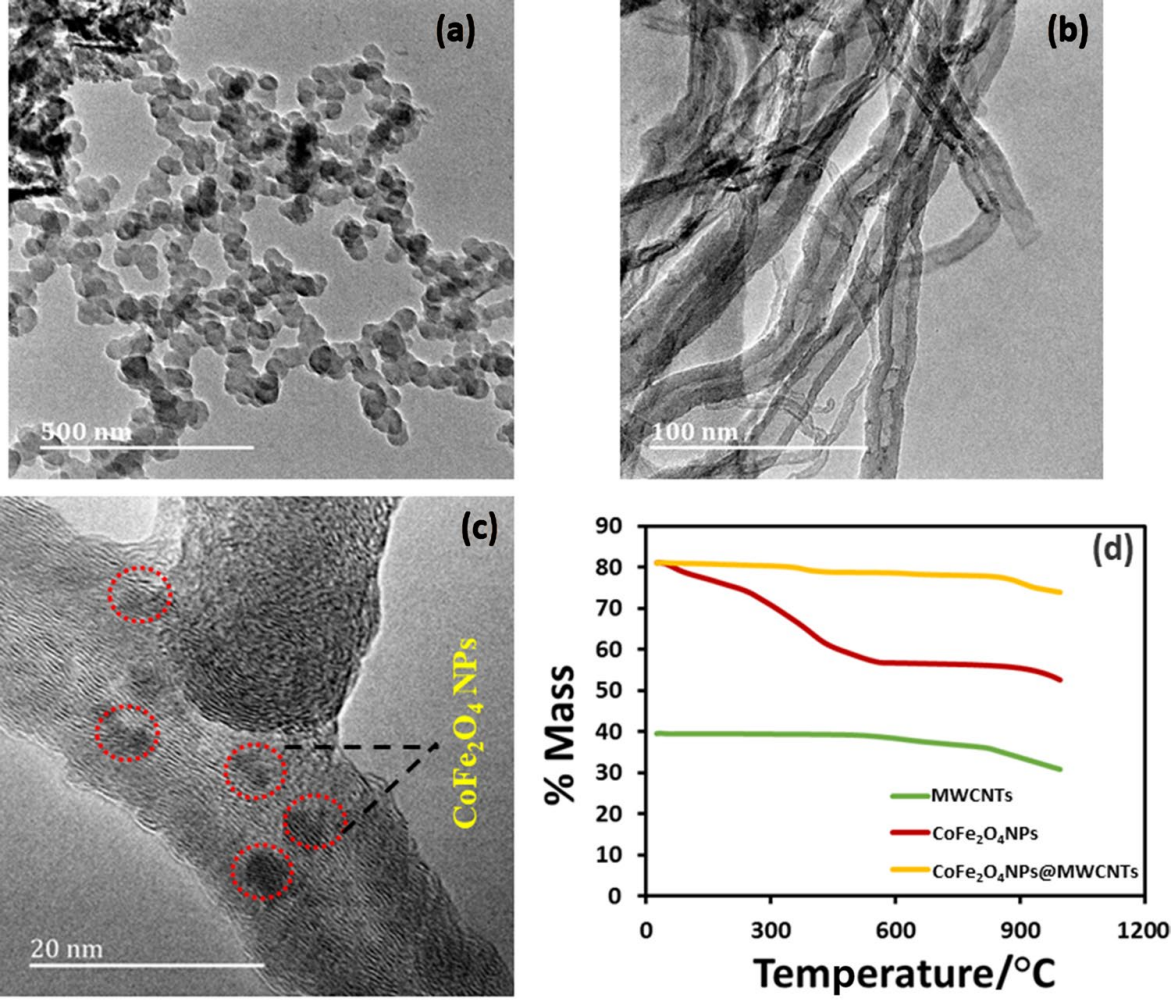
TEM images of (**a**) pure CoFe_2_O_4_NPs (**b**) pure MWCNTs (**c**) CoFe_2_O_4_NPs@MWCNTs and (**d**) TGA curves of (i) MWCNTs (ii) CoFe_2_O_4_NPs (iii) CoFe_2_O_4_NPs @MWCNTs.

FTIR spectra of CoFe_2_O_4_NPs and CoFe_2_O_4_NPs@MWCNTs were shown in Fig. 2a. The broad absorption band at 3,384–3,463 cm^−1^ corresponds to the O- H stretching vibrations of free or absorbed moisture. The bending of the hydroxyl group takes place approximately at1700 cm^−1^^25^. The characteristic absorption bands at 550 cm^−1^, 529 cm^−1^, 598 cm^−1^, 684 cm^−1^ attributed to the stretching vibration modes of the metal–Oxygen (Fe–O and Co–O) bonds in their respective tetrahedral and octahedral sites^26,27^. The specific characteristic bands at 2,859 cm^−1^, 1541 cm^−1^, and 1,345 cm^−1^ represented in both CoFe_2_O_4_NPs and CoFe_2_O_4_NPs@MWCNTs spectra are related to the asymmetric and symmetric stretching of COO, C=C and C–H of MWCNTs. Furthermore, FTIR is used to evaluate the spinel phase formation of CoFe_2_O_4_NPs. It demonstrate the position of the bivalent and trivalent metal ions present in the spinel structure and the vibration mode of the bond between oxygen atoms and the metal ions in the tetrahedral and octahedral sites. The crystal structure of NPs was further characterized using XRD. Figure 2b shows the XRD patterns of the CoFe_2_O_4_NPs and illustrates the characteristic (220), (222), (400), (311), (422) and (533) diffraction peaks of CoFe_2_O_4_NPs phase with FCC crystal structure. The normal crystallite size of CoFe_2_O_4_NPs was calculated from XRD patterns using Scherrer formula (d = 0.9 $\lambda$/β cos θ_B_)^25^. The TEM analysis was performed to assess the exact particle size of CoFe_2_O_4_NPs. As shown in Fig. 3a, the measured particle size was found to be in the range of 8–25 nm, which is in the agreement with XRD data. The obtained TEM data shows that the most of the particles are spherical in shape with monodispersity (see Fig. 3a). Figure 3b shows the pure MWCNTs with a tubular network like structure. Figure 3c illustrates the decoration of CoFe_2_O_4_NPs on the surface of the MWCNTs. Thermal behaviour of the CoFe_2_O_4_NPs@MWCNTs was investigated with TGA–DSC analysis. The TGA curve of MWCNTs showed well definite weight loss at 800 °C maybe due to the carbon oxidation^28^. In the case of CoFe_2_O_4_NPs two weight loss steps were observed. In the first step, an endothermic peak was observed in DTA curve with maximum peak at approximately 275 °C attributed to − 10.02% weight loss of moisture from the surface of CoFe_2_O_4_NPs. The second weight loss occurred at approximately 550 °C with a weight loss of − 3.20% due to the combustion of oleic acid surfactant. The profile of CoFe_2_O_4_NPs@MWCNTs, an additional weight loss at 350 °C, which can be attributed to the attached heavy ions (see Fig. 3d)^29^.

### Evaluation of electrode surface area

**Figure 4:**
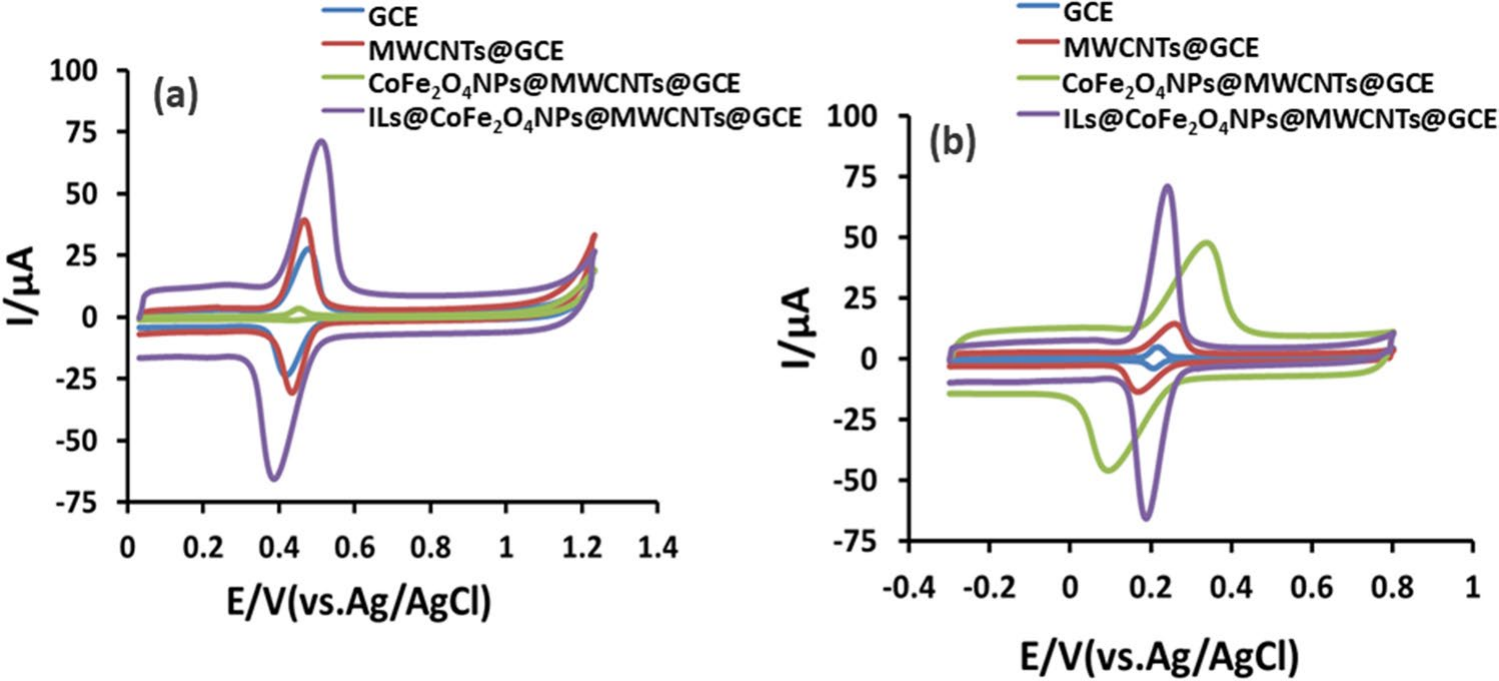
Cyclic Voltammograms of 0.1 mM (**a**) ETB and (**b**) PZM at (i) bare GCE (ii) MWCNTs@GCE (iii) CoFe_2_O_4_NPs@MWCNTs@GCE and (iv) IL@CoFe_2_O_4_NPs@MWCNTs@GCE.

Cyclic voltammetry is one of the versatile method used to monitor the behaviour of the fabricated working electrode. In this study, the effective surface areas of bare GCE, MWCNTs@GCE, and IL@CoFe_2_O_4_NPs@MWCNTs@GCE were investigated using voltammogram at a scan rate of 0.01 m Vs^−1^ (Fig. 4a,b). The obtained results demonstrated that the IL@CoFe_2_O_4_NPs@MWCNTs@GCE exhibited the amplified electrochemical signals due to the rapid conductivity of the electrons compared to that of MWCNTs@GCE and bare GCE. Therefore, the active surface area was measured using Randles–Sevcik Equation^30,31^.

1 $$ i_{pa} = 2.69 \times 10^{5} AC_{0} n^{3/2} D_{R}^{1/2} v^{1/2} $$

where $$ i_{pa}$$ is the anodic peak current, n is the number of electrons transferred, C_0_ is the concentration of PZM and ETB, D_R_ is the diffusion coefficient, A is the surface area of the electrode, and V is the scan rate. Based on the Eq. (1), the measured surface areas of IL@CoFe_2_O_4_NPs@MWCNTs@GCE, MWCNTs@GCE and bare GCE surfaces were found to be 10.32, 5.68 and 3.14 mm^2^ respectively. These results suggested that the surface area of the IL@CoFe_2_O_4_NPs@MWCNTs@GCE is 9-folds greater than MWCNTs@GCE and bare GCE, resulted in the enhanced electrocatalytic property.

### Influence of pH

**Figure 5:**
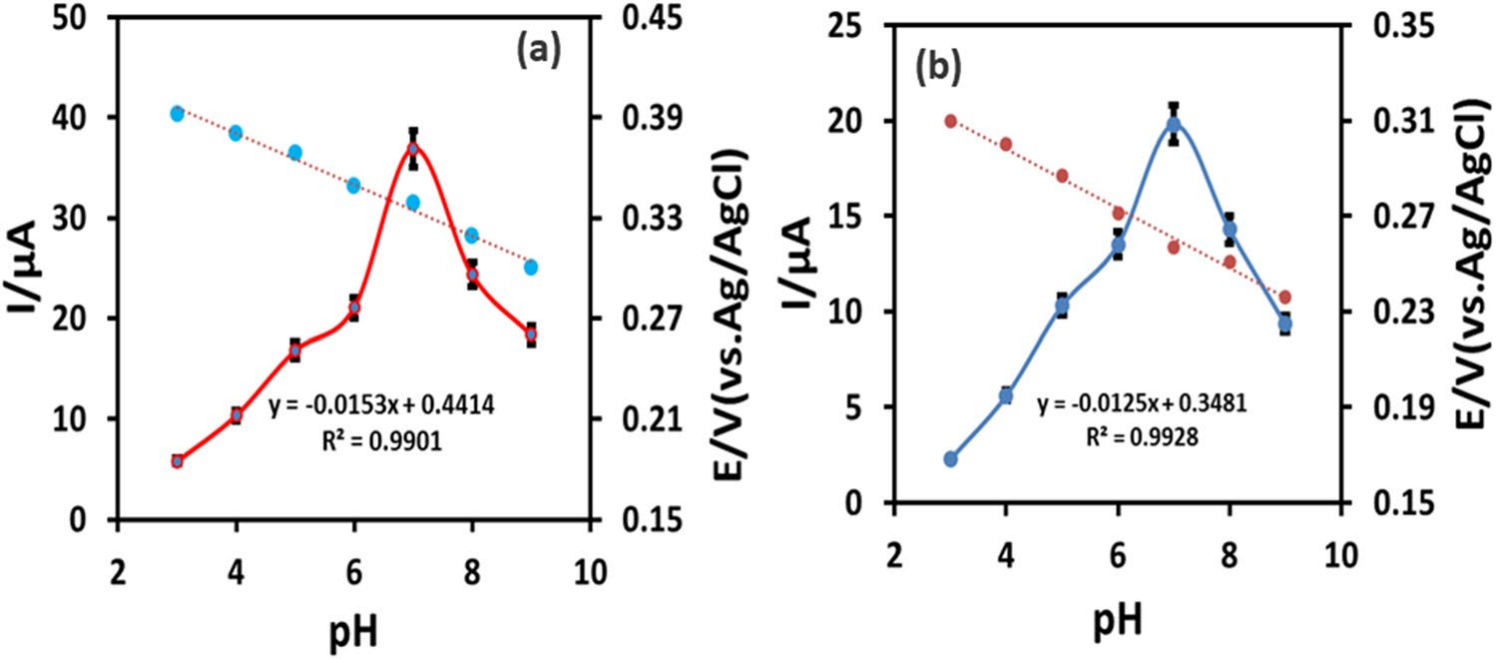
Effect of pH on the peak currents and peak potential of (**a**) ETB. (**b**) PZM.

The influence of the pH on the peak currents of ETB and PZM were examined in the pH range of 3.0–9.0 at the IL@CoFe_2_O_4_NPs@MWCNTs@GCE (see Fig. 5a,b).

The response current of the ETB and PZM was increased with increasing pH from 3.0 to 7.0. Beyond pH 7.0, the response current was further decreased. Therefore, pH 7.0 was selected as an optimum throughout the study. As depicted in Fig. 5a,b the peak potentials of ETB and PZM shifted towards positive potential with the increase in pH, indicating the direct involvement of protons redox reaction procedures. The linear regression equations of ETB and PZM are stated as follows individually:

2 $$ E = - 0.0{\text{153 pH }} + 0.{\text{4414 R}}^{{2}} = 0.{99}0{1} $$

3 $$ E = - 0.0{\text{125 pH }} + 0.{\text{3481 R}}^{{2}} = 0.{9928} $$

The obtained slopes from the current vs pH in the pH range of 3.0–9.0 indicates equal number of protons and electrons are participating in the reduction of ETB and PZM as described previously^32^.

### Deposition time

**Figure 6:**
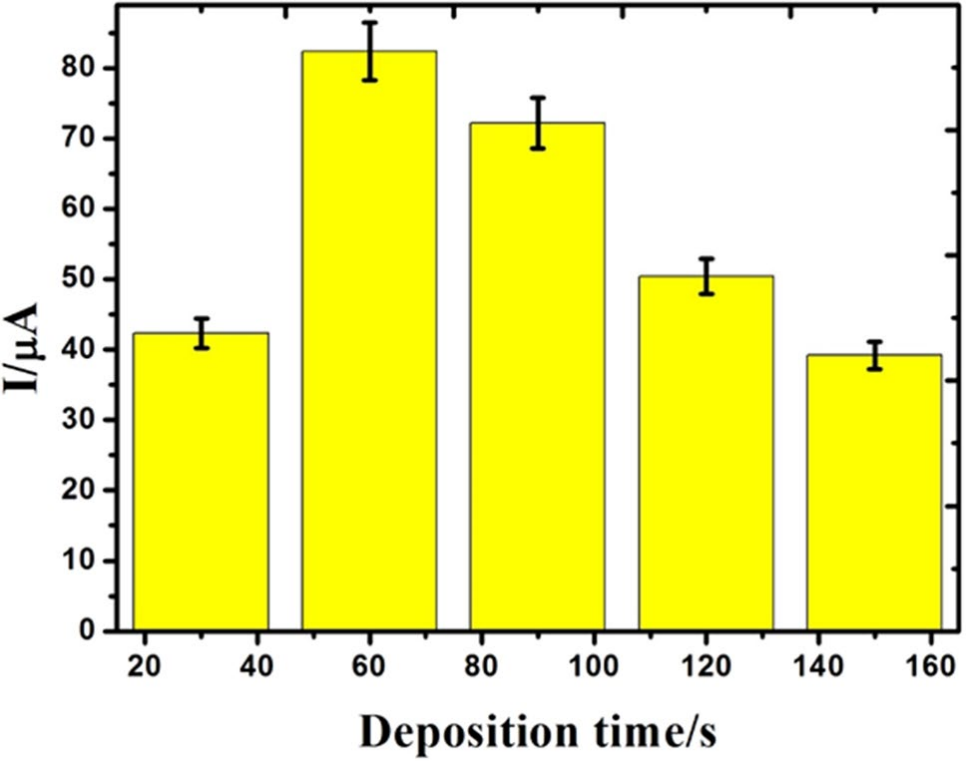
Peak current responses vs different deposition time with ranges of 30, 60, 90, 120 and 150 s.

Figure 6 represents the effect of deposition time as a function of response current from 30–150 s. It was noted that the anodic and cathodic peak currents were increased proportionally with the deposition time between 30 and 150 s. It is well known that the sensitivity improves with a longer deposition time due to the availability of electrode surface to the analyte at the lower concentration, however upper detection limits also increases due to the attainment saturation of the electrode surface at higher concentration of the analyte. In this study, the maximum response current was observed at 60 s and the peak current decreases gradually with the increase in the deposition time due to the achievement of saturation of GCE. By considering the sensitivity factor in to account, the optimum deposition time of 60 s was selected throughout the experiment.

### Simultaneous detection of ETB and PZM

**Figure 7:**
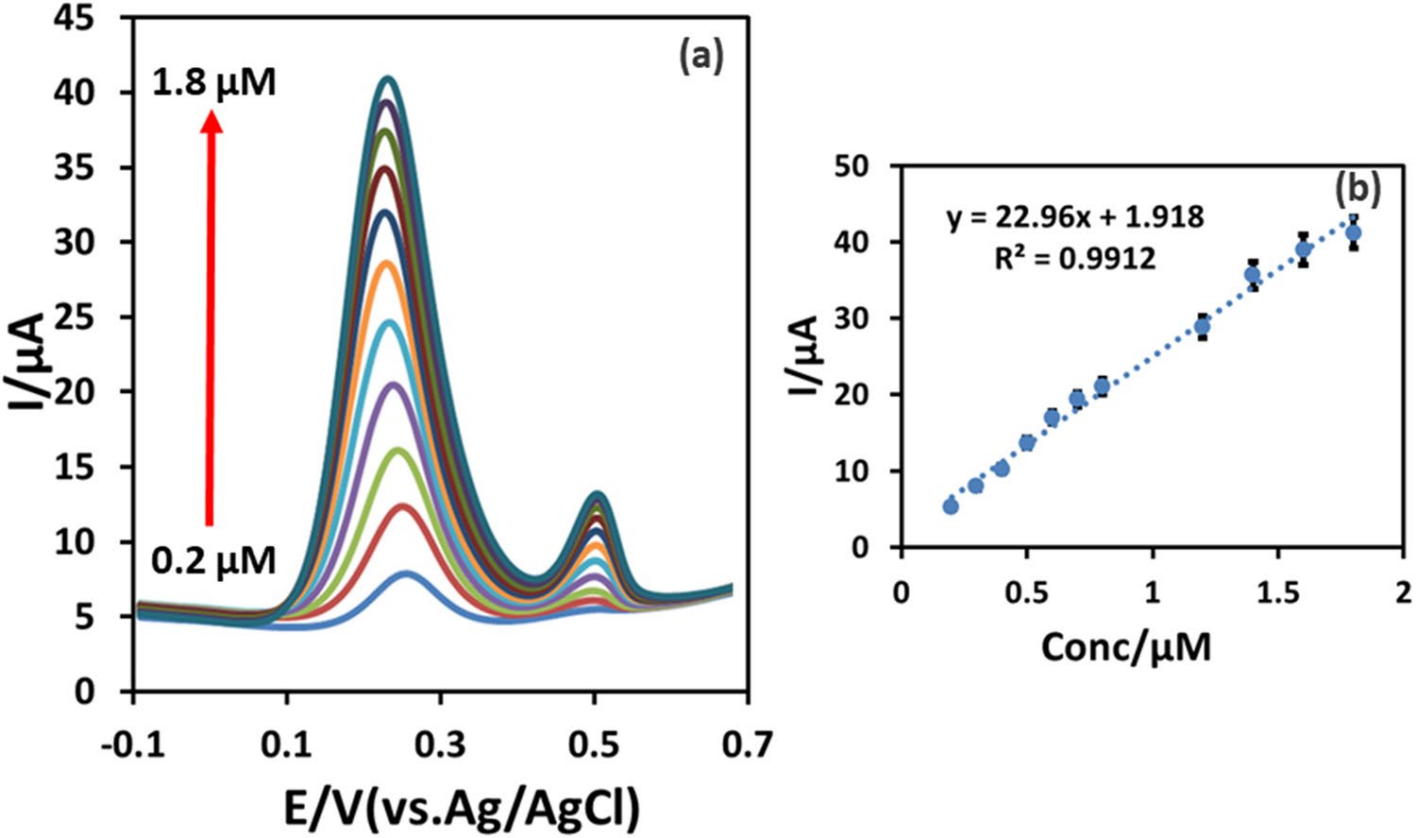
(**a**) Individual detection of ETB (0.2–1.8 µM) at fixed concentration of 0.1 µM PZM using DPV. (**b**) Calibration plot of the voltammetric currents as a function of the analyte concentrations.

**Figure 8:**
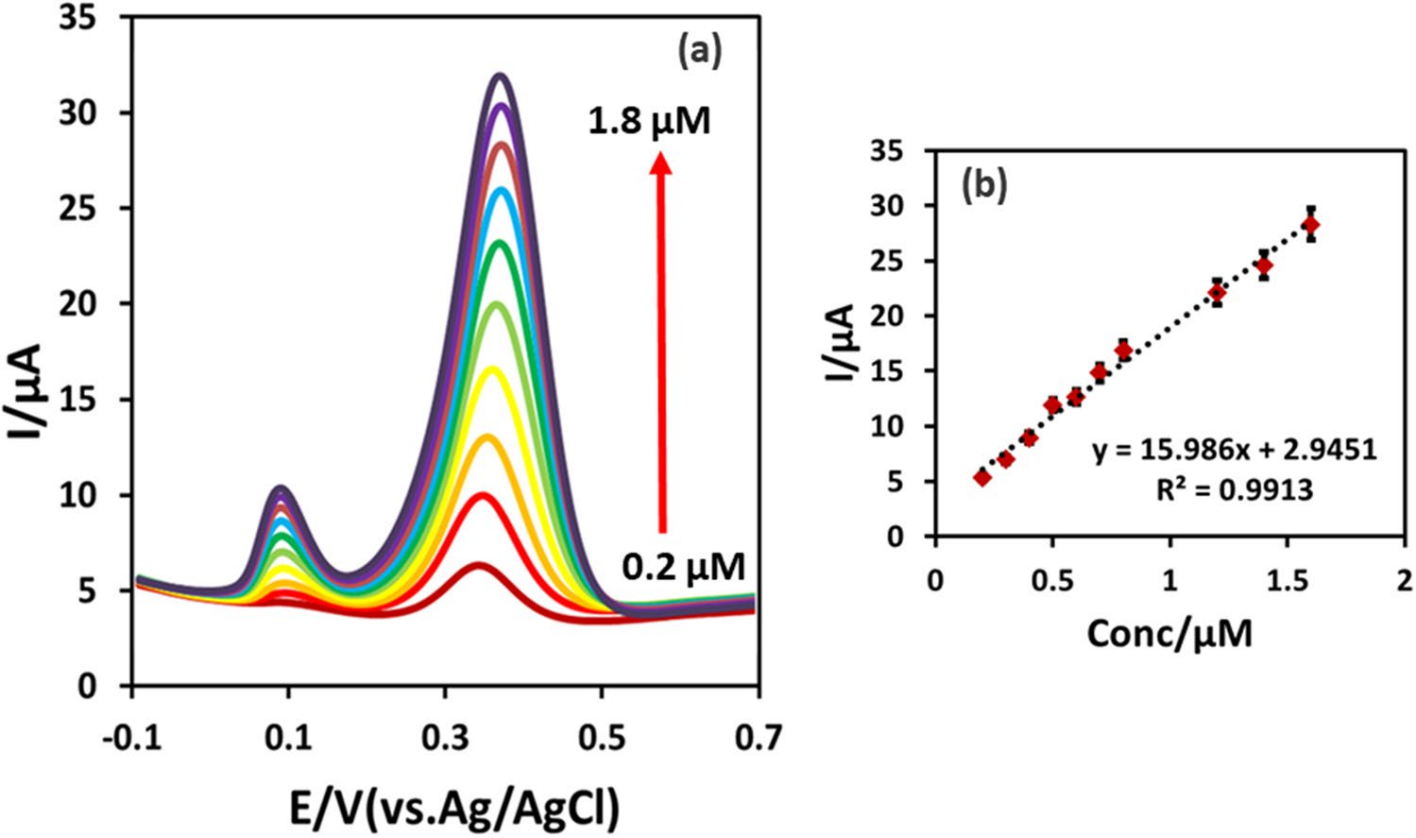
(**a**) Individual detection of PZM (0.2–1.8 µM) at fixed concentration of 0.1 µM ETB using DPV. (**b**) Calibration plot of the voltammetric currents as a function of the analyte concentrations.

**Figure 9:**
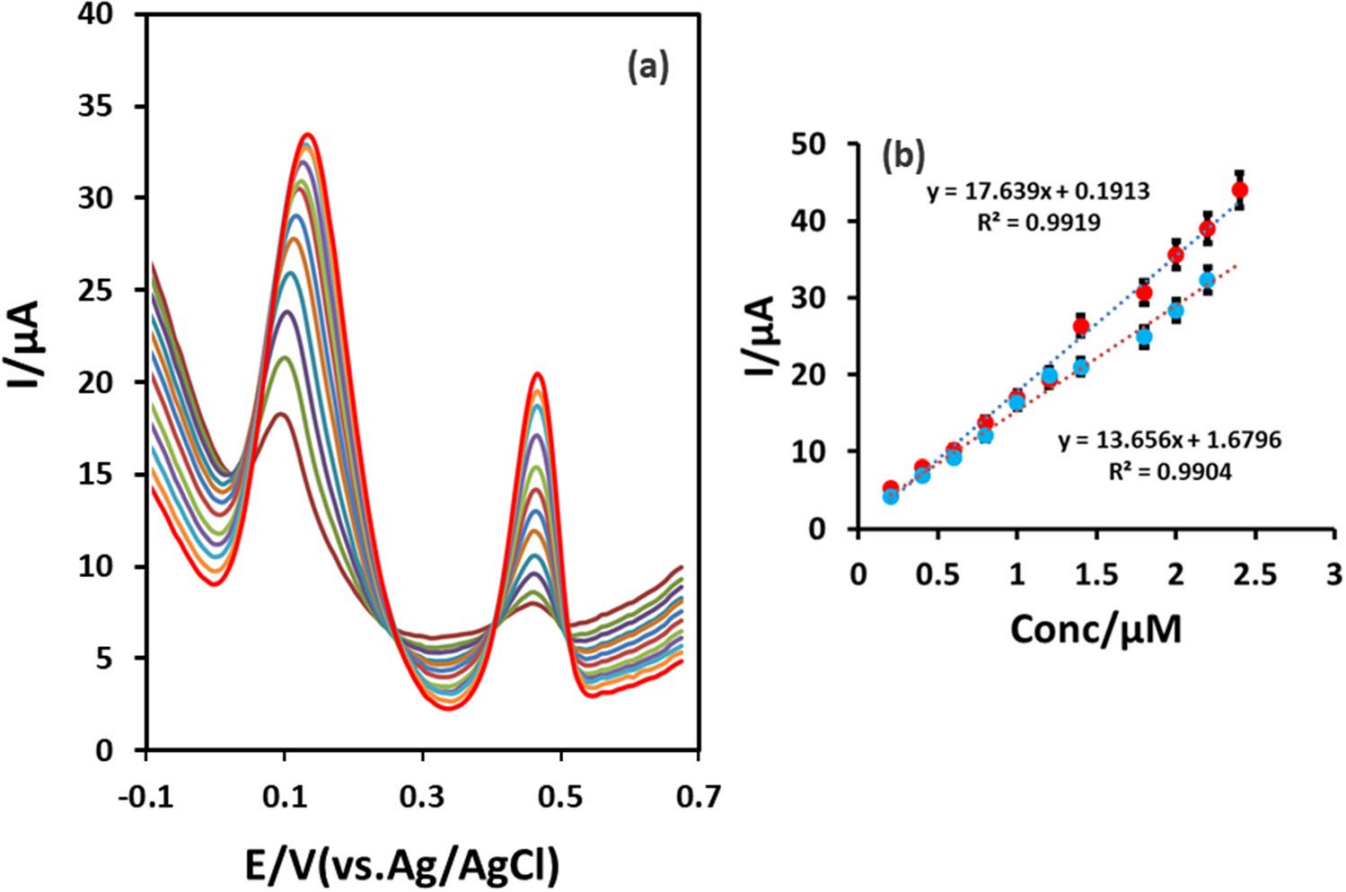
(**a**) Simultaneous detection of ETB (0.2–2.2 µM) and PZM (0.6–2.8 µM) with IL@CoFe_2_O_4_@MWCNTs@GCE using DPV (**b**) Calibration plot of the voltammetric currents as a function of the analyte concentrations.

Under the optimal experimental conditions, the analytical performance of the developed IL@CoFe_2_O_4_NPs@MWCNTs@GCE was assessed using DPV for the individual and as well as simultaneous detection of ETB and PZM. Firstly, for the individual detection of ETB and PZM, the concentration of ETB was fixed at 0.1 µM while concentration of PZM was changed from 0.2 to 1.8 µM (Fig. 7a,b) and vice-versa. The obtained results were depicted in Figs. 7a and 8a,b. Figures 7a and 8a showed that the increase in the concentration of ETB, the current has increased linearly with the unaffected peak current of PZM. Moreover, the calibration curve for ETB and PZM were noted as *I*_*pa*_ = 22.96ETB + 1.918 (*R*^*2*^ = 0.9912) and *I*_*pa*_ = 15.986PZM + 2.9451 (*R*^*2*^ = 0.9913). Secondly, for the simultaneous detection, the concentration of the ETB and PZM were concurrently increased, resulting in the linear increase in the peak current ranging from 0.2 to 2.2 µM for ETB and 0.6 to 2.8 µM for PZM as illustrated in Fig. 9. The linear regression equations were shown below:

4 $$ {\text{ETB}}I_{pa} = {17}.{\text{369ETB}} + 0.{1913}R^{2} = 0.{9919} $$

5 $$ {\text{PZM}}I_{pa} = {13}.{\text{656PZM}} + {1}.{6796}R^{2} = 0.{99}0{4} $$

In the case of individual and simultaneous detection of ETB and PZM, the slopes of linear regression equations were identical as shown in Fig. 9. Moreover, no significant interference effect was observed for the simultaneous detection of RTB and PZM due to the enhanced selectivity of developed IL@CoFe_2_O_4_NPs@MWCNTs@GCE sensor. Additionally, the LOD’s for ETB and PZM were individually and noted as 0.0201 and 0.010 μM respectively. The comparison of present sensor with the reported sensors were tabulated in Table 1.

**Table 1:** Comparison of analytical performance of reported sensors with an IL@CoFe_2_O_4_NPs@MWCNTs@GCE for ETB and PZM.

Electrode	Technique	Detection limit (μM)	Citation
**ETB**			
Au-polyvinylpyrrolidone-poly (8-anilino-1-naphthalene sulphonic acid)-Ag-cytochrome P450-2E1	CV and DPV	0.7	Ref.^33^
Nafion-multiwalled carbon nanotubes-Screen printed carbon electrode	CV and SWV	8.4	Ref.^35^
Gold microelectrode	CV and SWV	4.73	Ref.^34^
Carbon electrode	FIA	100	Ref.^36^
Tyrosine-glassy carbon electrode	CV and DPV	9.61	Ref.^16^
Platinum electrode	CE	24.2	Ref.^37^
Harsh radish peroxidase-Zinc oxide nanoparticles-Reduced graphene oxide-Glassy carbon electrode	CV and DPV	0.0214	Ref.^38^
IL@CoFe_2_O_4_NPs@MWCNTs@GCE	CV and DPV	0.0201	This work
**PZM**			
Cytochrome c-copper oxide nanoparticles-multiwalled carbon nanotubes-glassy carbon electrode	CV and DPV	0.0038	Ref.^39^
Graphene oxide-poly arginine poly-l-methionine-glassy carbon electrode	CV and DPV	3.28	Ref.^40^
Poly-l-methionine-glassy carbon electrode	CV and DPV	0.035	Ref.^41^
Screen printed carbon electrode-poly-histidine prepared by histidine monomer electro polymerization	DPV and SWV	68	Ref.^42^
Graphene-zinc oxide nanoparticles-carbon paste electrode	CV and DPV	0.0431	Ref.^43^
Poly-l-methionine-reduced graphene oxide-glassy carbon electrode	CV and DPV	0.16	Ref.^44^
IL@CoFe_2_O_4_NPs@MWCNTs@GCE	CV and DPV	0.010	This work

*CV* cyclic voltammetry, *DPV* differential pulse voltammetry, *SWV* squarewave voltammetry, *FIA* flow injection analysis, *CE* capillary electrophoresis.

### Reproducibility, stability and interferences

The performance of the IL@CoFe_2_O_4_NPs@MWCNTs@GCE was evaluated with DPV under the optimized conditions (pH: 7.0, accumulation time: 100 s and suspension volume: 5 μL). The reproducibility of the developed IL@CoFe_2_O_4_NPs@MWCNTs@GCE examined by using the same electrode modification for six individual measurements with 0.1 μM of ETB and PZM. The results obtained shows that the relative standard deviation (RSD) was 2.62%, indicating the acceptable reproducibility for the detection of ETB and PZM. To validate the stability, the IL@CoFe_2_O_4_NPs@MWCNTs@GCE was kept at ambient temperature in the laboratory for 15 days and then the electrochemical measurements were performed. Interestingly, it was found that 95% of the electrochemical signals were retained. The interference effect was studied by including organic compounds, common ions and electrochemical signals were noted with DPV at 0.1 μM ETB and PZM. The obtained results were presented in Table 2 demonstrated that the 50-fold increase in the concentration of ions such as Ca^2+^, K^+^ , Mg^2+^, Na^+^, Cu^2+^, Cl^−^, NO^3−^, Cd^2+^, and SO_4_^2−^ does not interfere with the ETB and PZM signals. On the other hand, 100-fold increase in the concentration of ascorbic acid, glucose and uric acid also does not impact the detection of ETB and PZM.

**Table 2:** Influences of interferents on the electrochemical response of 0.1 μM ETB and PZM, as measured by DPV with an IL@CoFe_2_O_4_NPs@MWCNTs@GCE.

Interferences	Interferences concentration (μM)	Recovery (%)^a^
Ca^2+^	50	99.2
K^+^	50	96.1
Mg^2+^	50	99.6
Na^+^	50	98.4
Cu^2+^	50	101.2
Cl^−^	50	99.3
NO^3−^	50	100.1
Cd^2+^	50	99.1
Ascorbic acid	100	102.4
Uric acid	100	101.6
Glucose	100	104.2
Lactoferrin	50	96.2
Transferrin	50	95.8
Human serum albumin	50	96.8
Bovine serum albumin	50	96.6

^a^n = five individual determinations.

### Real sample analysis

The real applicability of IL@CoFe_2_O_4_NPs@MWCNTs@GCE was examined by considering the pharmaceutical formulations of ETB and PZM. The standard addition procedure was adopted for the detection of ETB and PZM and the obtained results were tabulated in Table 3. The acceptable recoveries were noted for the detection of ETB and PZM. The developed IL@CoFe_2_O_4_NPs@MWCNTs@GCE sensor might be competent in the detection of ETB and PZM in more commercial pharmaceuticals compared to that of other related techniques^16,33–44^.

**Table 3:** Detection of ETB and PZM in commercial pharmaceutical samples with an IL@CoFe_2_O_4_NPs@MWCNTs@GCE using DPV.

Sample	Addition (μM)	Found (μM)	RSD^b^ (%)	Recovery (%)^a^
ETB	2	1.93	1.86	96.5
	4	4.12	2.62	103
	6	5.92	2.30	98.6
PZM	2	2.04	1.91	102
	4	3.89	2.36	97.2
	6	6.10	2.61	101.6

^a^n = five individual determinations.

^b^Relative standard deviation (n = 5).

## Conclusions

We report, for the first time, simultaneous electrochemical detection of ETB and PZM with IL@CoFe_2_O_4_NPs@MWCNTs in pharmaceutical formulations more effectively. The investigated drugs are formulated together in a single pharmaceutical dosage procedures. In addition, these results confirmed that the IL@CoFe_2_O_4_NPs@MWCNTs is highly selectivity, sensitivity and low detection limits for the simultaneous detection strategy due to the high surface area and rapid electron conductivity of CoFe_2_O_4_NPs. Finally, the capability of the developed electrochemical sensor was tested on pharmaceutical formulations, yielding a good analytical performance and thus providing a promising alternative for sensing applications in the pharmaceutical and biochemical industries.
